# Mental health among sexually and gender diverse adolescents in Indonesia and Vietnam: Results from the National Adolescent Mental Health Surveys

**DOI:** 10.1186/s13034-025-00921-5

**Published:** 2025-07-31

**Authors:** Shoshanna L. Fine, Astha Ramaiya, Mengmeng Li, Amirah Ellyza Wahdi, Siswanto Agus Wilopo, Vu Manh Loi, Nguyen Duc Vinh, Yohannes Dibaba Wado, Joemer C. Maravilla, James G. Scott, Holly E. Erskine, Robert Wm Blum

**Affiliations:** 1 Department of Population, Family and Reproductive Health, Johns Hopkins Bloomberg School of Public Health, Johns Hopkins University, Baltimore, MD USA; 2 Department of Mental Health, Johns Hopkins Bloomberg School of Public Health, Johns Hopkins University, Baltimore, MD USA; 3 Center for Reproductive Health, Faculty of Medicine, Public Health, and Nursing, Universitas Gadjah Mada, Yogyakarta, Indonesia; 4 Department of Biostatistics, Epidemiology, and Population Health, Faculty of Medicine, Public Health, and Nursing, Universitas Gadjah Mada, Yogyakarta, Indonesia; 5 Institute of Sociology, Vietnam Academy of Social Sciences, Hanoi, Vietnam; 6 African Population and Health Research Center, Nairobi, Kenya; 7 School of Public Health, The University of Queensland, Herston, QLD Australia; 8 Queensland Centre for Mental Health Research, Wacol, QLD Australia; 9 Institute of Health Sciences and Nursing, Far Eastern University, Manila, Philippines; 10 Child Health Research Centre, The University of Queensland, South Brisbane, QLD Australia; 11 Child and Youth Mental Health Service, Children’s Health Queensland, South Brisbane, QLD Australia; 12 Institute for Health Metrics and Evaluation, University of Washington, Seattle, WA USA

**Keywords:** LGBT, Sexual orientation, Gender identity, Mental health, Adolescent, Indonesia, Vietnam

## Abstract

**Background:**

Sexually and gender diverse adolescents worldwide are at heightened risk of experiencing mental health issues, including depression, anxiety, and suicidal behaviours. To date, however, no studies have used nationally representative data to explore the prevalence of sexual and gender diversity and associated mental health issues among adolescents in Indonesia and Vietnam.

**Methods:**

Data were drawn from the National Adolescent Mental Health Surveys, nationally representative household surveys of mental health among adolescents aged 10–17 years in Indonesia and Vietnam. Questions about gender identity and romantic/sexual attraction were only asked to those aged 12–17. Weighted logistic regression analyses were used to assess the associations between (1) gender identity and (2) sexual orientation and past 12-month depressive symptoms, anxiety symptoms, and suicidal ideation.

**Results:**

Of 4,300 adolescents in the Indonesia sample and 4,376 adolescents in the Vietnam sample, 5.3% and 5.9% respectively reported gender diverse identities, while 9.4% and 12.6% respectively reported sexually diverse orientations. In Indonesia, gender diverse adolescents had significantly increased odds of past 12-month depressive symptoms (aOR: 2.95; 95% CI: 1.17–4.35), anxiety symptoms (aOR: 1.88; 95% CI: 1.24–2.85), and suicidal ideation (aOR: 6.16; 95% CI: 2.73–13.89) as compared to their cisgender peers. Sexually diverse adolescents had significantly increased odds of depressive symptoms (aOR: 2.64; 95% CI: 1.51–4.62), anxiety symptoms (aOR: 2.02; 95% CI: 1.41–2.89), and suicidal ideation (aOR: 3.41; 95% CI: 1.25–9.29) as compared to their heterosexual peers. In Vietnam, gender diverse adolescents only had increased odds of anxiety symptoms (aOR: 1.58; 95% CI: 1.13–2.22), whereas sexually diverse adolescents had increased odds of depressive symptoms (aOR: 2.58; 95% CI: 1.36–4.92), anxiety symptoms (aOR: 1.58; 95% CI: 1.14–2.19), and suicidal ideation (aOR: 2.84; 95% CI 1.13–7.14).

**Conclusions:**

In Indonesia and Vietnam, a considerable proportion of adolescents report gender diverse identities or sexually diverse orientations. Given that sexually and gender diverse adolescents are at increased risk of depression, anxiety, and suicidal ideation, it is critical that appropriate, culturally safe mental health and psychosocial support services are available to provide targeted assistance to these young people.

**Supplementary Information:**

The online version contains supplementary material available at 10.1186/s13034-025-00921-5.

## Background

Mental disorders are a leading cause of health-related disability among adolescents, affecting an estimated 14% of adolescents globally [1]. A large body of research has consistently demonstrated that sexually diverse adolescents– including those who identify as lesbian, gay, bisexual, queer, or pansexual– experience increased risks for poor mental health [2]. For example, one meta-analysis of 23 population-based studies across eight countries (largely concentrated in North America or Europe) found that sexually diverse adolescents have approximately three times the odds of depressive symptoms compared to their heterosexual peers [3]. Other meta-analyses have uncovered similarly high odds of suicidal ideation and suicide attempts among sexually diverse adolescents [4–6], and studies from around the world have also found that these adolescents report elevated rates of other mental health issues including anxiety, disordered eating, substance abuse, and self-harm [2, 5, 7–10].

Similar patterns have been documented for gender diverse adolescents, defined as those whose gender identity does not match their sex assigned at birth or who do not identify with the gender binary [11]. This may include adolescents who identify as transgender, non-binary, gender non-conforming, gender-queer, or gender-fluid. While evidence in this area is still emerging, recent large-scale studies in China [12], Thailand [13], Canada [14, 15], the United Kingdom [16], and the United States [17, 18] have found substantially increased risks of depression, anxiety, substance abuse, suicide, and self-harm among gender diverse adolescents compared to their cisgender peers. Existing research has also suggested that there may be important disparities within this population (e.g., between those assigned female at birth compared to those assigned male at birth), although such variations are marked by notable geographic inconsistencies [12, 18].

It is important to emphasize that sexual orientation and gender identity are distinct constructs. That said, similar theoretical frameworks have been used to explain the marked mental health disparities which consistently emerge between sexually and gender diverse (SGD) adolescents and their heterosexual, cisgender peers. Most notably, the minority stress model suggests that societal marginalization of SGD individuals results in unique stressors, including stigma, discrimination, and victimization, which increase their vulnerability to adverse mental health outcomes [19, 20]. Many studies have provided support for this model among SGD adolescents living in diverse settings around the world. SGD adolescents have an increased risk of experiencing bullying victimization, family maltreatment, structural discrimination, and internalized homophobia or transphobia, and these minority stressors have repeatedly been identified as key drivers of mental health issues within this population [21–23]. In addition, beyond the traditional minority stress model, researchers have suggested that mental health issues may arise among SGD adolescents due to their unique experiences with navigating identity exploration, solidification, and acceptance during this critical developmental period [24].

Importantly, the majority of existing studies focused on SGD adolescents have been conducted in high-income countries, despite approximately 90% of the world’s adolescents living in low- and middle-income countries (LMICs) [25]. This represents a significant gap in our understanding of challenges facing these young people, as evidence suggests that SGD adolescents living in LMICs may experience particular risks as a result of increased stigma, discrimination, violence, and harassment in these settings [26, 27]. Such risks may be further exacerbated by national restrictions: a disproportionate number of LMICs globally have laws and policies that criminalize SGD behaviours, including, in some settings, life imprisonment or the death penalty [28]. The extent of this issue remains unknown, however, due to the lack of reliable data capturing the prevalence, characteristics, and mental health issues of SGD adolescents living in LMICs.

These research gaps are evident in both Indonesia and Vietnam, where the current study was carried out. Of 25 studies identified in a recent systematic review of the prevalence and correlates of mental health issues among SGD populations in Southeast Asia, only one included Indonesia alongside Vietnam, Malaysia, Myanmar, and Thailand, and only two focused exclusively on Vietnam [29]. The multi-country study of 3,240 university students suggested that there may be higher rates of depression, suicide attempts, and substance abuse among those with SGD identities; however, the combined nature of these results precludes any country-specific conclusions [30]. A more contemporary study in Indonesia focused on an online sample of 5,211 adults from across the country in the context of the COVID-19 pandemic, and found increased odds of self-harm and suicidal ideation among those who identified as sexually diverse or gender diverse [31]. In another study of 363 men who have sex with men (MSM) and transgender people in Bali, the majority (69.1%) reported moderate to severe levels of psychological distress, which was associated with experiences of stigma and discrimination [32]. To our knowledge, however, there are no existing studies that explore these issues among Indonesian adolescents more broadly.

In Vietnam, the most relevant existing study included a representative sample of 6,363 young people aged 15–22 living in Hanoi. Among approximately 4% who identified as sexually diverse, there was no evidence for increased odds of suicidal ideation or suicide attempts [33]. The authors suggested that Confucian values in this context may be protective against suicide, although they also acknowledged that the very small size of the subgroup identifying as sexually diverse and reporting suicidality may have been insufficient for detecting any association. By contrast, a study focused on 1,936 sexually diverse women found that 16.8% reported one or more lifetime suicide attempts, with experiences of family rejection associated with higher odds of attempted suicide [34]. Family rejection was also associated with lower life satisfaction, more depressive symptoms, and increased alcohol use in this population. Additional studies have documented an elevated burden of depression, anxiety, post-traumatic stress disorder, suicidal ideation, and substance abuse among Vietnamese MSM [35–37]. Again, very little existing research has focused on Vietnamese adolescents, and there are also few studies that include gender diverse populations.

The current study addresses these gaps in evidence by reporting the prevalence of sexual and gender diversity and associated mental health issues among adolescents using data from the National Adolescent Mental Health Surveys (NAMHS) [38] in Indonesia and Vietnam. Specific aims include the following: (1) describe the prevalence of sexual and gender diversity among adolescents in Indonesia and Vietnam; (2) describe the differences in sociodemographic characteristics and mental health issues among SGD adolescents in both countries; and (3) explore the associations between sexual and gender diversity and depressive symptoms, anxiety symptoms, and suicidal ideation among SGD adolescents in both countries compared to their heterosexual, cisgender peers. These mental health issues were selected due to significant empirical evidence regarding their salience among SGD adolescents globally [2], as well as their specific relevance among adolescents in Indonesia and Vietnam [39].

## Methods

### Study design

Data were drawn from NAMHS, nationally representative household surveys of mental health among adolescents in Indonesia (I-NAMHS) and Vietnam (V-NAMHS). Detailed methodology for NAMHS in each country have been published elsewhere [38, 39]. In brief, a multi-stage stratified cluster sampling design was used in each country to select a nationally representative, household-based sample of adolescents aged 10–17 years and their primary caregiver. Adolescents were excluded if they lived in the household less than 50% of the time, if they were married or living independently (i.e., without a primary caregiver), if they or their primary caregiver did not speak the language required by the survey (Bahasa Indonesia for I-NAMHS and Vietnamese for V-NAMHS), or if they were unable to participate due to severe physical or cognitive impairment. Informed written consent and assent were required from primary caregivers and adolescents, respectively. Data were collected in-person by trained interviewers between March and November 2021 in Indonesia and between September and December 2021 in Vietnam. Interviewers were trained to follow a structured protocol in the event of participant distress (including disclosure of imminent risk of harm), and all participants were given an information sheet including available mental health services in their area. The final sample consisted of 5,664 adolescents from 34 provinces in Indonesia (92.2% response rate), and 5,996 adolescents from 38 provinces in Vietnam (81.1% response rate). As only those aged 12–17 years were asked about gender identity and romantic/sexual attraction, the current study is limited to 4,300 adolescents in Indonesia and 4,376 adolescents in Vietnam.

### Study settings

*Indonesia.* Comprised of approximately 17,500 islands, Indonesia is characterized by immense ethnic, cultural, and linguistic diversity. It is the fourth most populated country in the world, with a population of nearly 280 million people, of which 17% are adolescents [40]. It is also the largest Muslim majority country, with around 87.5% of the population identifying as Muslim [41]. While there is evidence for sexual and gender diversity as important aspects of traditional cultures across different parts of the Indonesian archipelago [42], the current environment is marked by increasing conservatism and intolerance towards SGD populations [43]. Although there are no explicit laws at the national level criminalizing SGD identities or behaviours, country-wide laws and policies generally do not recognize or support the rights of SGD individuals [28, 42]. Further, there are several provinces and cities which prohibit same-sex intimacy according to local ordinances [28]. This includes Aceh, which is the only Indonesian province to apply Sharia law.

*Vietnam.* A country of approximately 100 million people, of which 14% are adolescents, Vietnam has been marked by rapid socioeconomic development since its adoption of *Doi Moi* (“renovation”) and transition towards a market-oriented economy in 1986 [44, 45]. During the subsequent decade, mounting government concerns around negative Western influences– compounded by the HIV/AIDs epidemic– deepened social stigma regarding drug use, sex work, and homosexuality [46–48]. Contemporary Vietnam has a growing SGD rights movement, including considerable discussion at the national level around the legalization of same-sex marriage as well as the current legalization of gender reassignment surgery for transgender individuals [47, 49]. For example, while Vietnam’s Marriage and Family Law of 2000 banned same-sex marriage, this law was amended in 2014 to decriminalize same-sex marriage, although such marriages are still not formally recognized by the state [46]. Despite such progress, widespread stigma and discrimination endure, with a number of studies suggesting that traditional Confucian values prioritizing filial piety, patriarchal gender norms, and communalism contribute to deeply entrenched homophobia and transphobia [50–52].

### Measures

The NAMHS instrument included sections administered separately to the primary caregiver and the adolescent, and captured information related to demographics, mental disorders, suicidal behaviours, and related risk and protective factors. In each country, the instrument was translated into the local language and then back-translated into English in order to ensure comparability of meaning. Additionally, the local language versions were reviewed by in-country mental health clinicians to confirm linguistic and cultural appropriateness. Prior to administration of the national surveys, the instrument was piloted in each country among approximately 50 households, with results used to inform changes to translations where necessary. More detailed information on the development and adaptation of the NAMHS instrument has been published elsewhere [38].

*Demographic variables.* Demographic variables included primary caregiver-reported sex and age. In addition, urbanicity was determined based on the household’s location according to classification by country-level statistical agencies. Household wealth, as measured by wealth tertiles, was generated using a principal component analysis of items from a standardized asset-based wealth index. Details regarding this analytic approach have been described previously [39].

*Gender identity.* Adolescents were asked to report on their own gender using the following question: “Which of the following best describes you?” Response options included “a boy,” “a girl,” “both a boy and a girl,” “other,” “don’t know,” and “prefer not to say.” Gender identity was assigned by comparing primary caregiver-reported sex with adolescent-reported gender, with adolescents categorized as “cisgender,” “transgender,” “non-binary,” “other,” or “uncertain.” Due to small cell sizes, these categories were further collapsed into (0) cisgender, (1) gender diverse (including transgender, non-binary, and other), or (2) uncertain. Adolescents were excluded if they endorsed “prefer not to say” for their gender identity (Indonesia: *n* = 79; Vietnam: *n* = 74).

*Sexual orientation.* Adolescents were asked to report on their romantic/sexual attraction using the following question: “Which of the following best describes who you are romantically attracted to? By attraction we mean having a crush on, wanting to have sex with, or being in love with someone.” Response options included “only boys,” “only girls,” “both boys and girls,” “neither boys nor girls,” “other,” “don’t know,” and “prefer not to say.” Sexual orientation was assigned by considering adolescent-reported gender alongside attraction, with adolescents categorized as “heterosexual,” “homosexual,” “bisexual,” “other,” “no attraction, or “uncertain.” For example, those who indicated that they were “a boy” and were attracted to “only boys” were categorized as homosexual. Adolescents who endorsed “both a boy and a girl” or “other” for their gender identity, and who indicated an attraction to “only boys” or “only girls,” were categorized as “other,” as were those who indicated an attraction to “other” regardless of their gender identity. Adolescents who endorsed “don’t know” for their attraction were categorized as “uncertain” regardless of their gender identity. These categories were further collapsed into (0) heterosexual, (1) sexually diverse (including homosexual, bisexual, and other), (2) no attraction, and (3) uncertain. Adolescents were excluded if they endorsed “prefer not to say” for their attraction, or if they endorsed “don’t know” or “prefer not to say” for their gender identity and indicated an attraction to “only boys” or “only girls” (Indonesia: n = 362; Vietnam: n = 331).

*Depressive symptoms.* Depressive symptoms were measured using the major depressive disorder module from the Diagnostic Interview Schedule for Children, Version 5 (DISC-5), a highly structured diagnostic tool for assessing mental disorders in children and adolescents which is designed to be administered by trained lay interviewers [53]. For the purposes of analysis, adolescents were considered to have heightened depressive symptoms if they met at least half of the symptoms required for past 12-month diagnosis of major depressive disorder according to the diagnostic requirements of the Diagnostic and Statistical Manual of Mental Disorders, Fifth Edition (DSM-5) [54]. This operationalization of “subthreshold” cases aligns with DISC-5 guidelines and has been used in prior preliminary analyses based off NAMHS data [55, 56]. While the DISC-5 is a diagnostic instrument, we chose to also include adolescents with subthreshold symptom levels given: (1) the low prevalence of mental disorders in both Indonesia (5.5%) and Vietnam (3.3%) according to NAMHS [39]; and (2) the relevance of considering targeted early intervention efforts for those with heightened symptoms which do not rise to the level of diagnosable psychopathology.

*Anxiety symptoms.* Anxiety symptoms were measured using the generalized anxiety disorder and social phobia modules from the DISC-5. As with depression, adolescents were considered to have heightened anxiety symptoms if they met at least half of the symptoms required for past 12-month diagnosis of generalized anxiety disorder and/or social phobia according to the diagnostic requirements of the DSM-5 [54]. We elected to combine information from across these two different modules due to the pertinence of considering general anxiety symptoms as opposed to specific categorical diagnoses.

*Suicidal ideation.* Adolescents were read a definition of suicide and then asked to report on past 12-month suicidal ideation using the following question: “During the past 12 months, have you ever thought about attempting suicide?” Adolescents could indicate either no (0) or yes (1) in response to this question. Adolescents were excluded if they endorsed “don’t know” or “prefer not to say” (Indonesia: *n* = 19; Vietnam: *n* = 48).

### Statistical analysis

In all analyses, appropriate population weights were used to account for the complex sampling design. First, weighted descriptive analyses were conducted to describe the prevalence of sexual and gender diversity among adolescents in each country. Rao-Scott chi-square tests [57] were used to compare the differences in sociodemographic characteristics and mental health issues across groups (by gender identity: cisgender, gender diverse, or uncertain; by sexual orientation: heterosexual, sexually diverse, no attraction, or uncertain). The Bonferroni correction was used to set the alpha level for comparisons. Second, a series of weighted logistic regression analyses were estimated to assess the associations between sexual and gender diversity and mental health issues in each country. Separate analyses were used to explore the relationship between gender identity (categorized as cisgender, gender diverse, or uncertain) and three separate mental health issues: past 12-month depressive symptoms, anxiety symptoms, and suicidal ideation. Equivalent analyses were used to explore the relationship between sexual orientation (categorized as heterosexual, sexually diverse, no attraction, or uncertain) and the same three mental health issues. In addition, supplementary analyses explored full diagnosis of major depressive disorder and anxiety disorders (generalized anxiety disorder, social phobia, and any anxiety disorder) according to DSM-5 criteria as outcome variables. All final models controlled for primary caregiver-reported sex, age, urbanicity, and wealth tertile. These specific sociodemographic covariates were selected based on robust evidence demonstrating their significant influence on mental health [58–61]. All analyses were undertaken using Stata 14.2 [62] and statistical significance was established at the 0.05 alpha level.

## Results

### Sociodemographic characteristics

The weighted sociodemographic characteristics and mental health issues of the sample across Indonesia and Vietnam are presented in Table 1.

**Table 1:** Sociodemographic characteristics and mental health issues by country

	Indonesia	Vietnam
	% (95% CI)	% (95% CI)
Primary caregiver-reported sex		
Male	50.5 (48.4–52.7)	51.9 (49.7–54.0)
Female	49.5 (47.3–51.6)	48.1 (46.0–50.3)
Age group		
12–14 years	53.9 (51.6–56.1)	51.9 (49.9–53.9)
15–17 years	46.1 (43.9–48.4)	48.1 (46.1–50.1)
Urbanicity		
Rural	30.8 (22.7–40.2)	67.8 (62.7–72.5)
Urban	69.2 (59.8–77.3)	32.2 (27.5–37.3)
Gender identity		
Cisgender	88.8 (86.6–90.7)	86.3 (82.8–89.1)
Transgender	2.6 (1.9–3.6)	2.6 (2.0–3.4)
Non-binary	2.3 (1.6–3.1)	2.5 (1.8–3.5)
Other	0.4 (0.2–0.7)	0.8 (0.4–1.3)
Uncertain	5.9 (4.7–7.5)	7.9 (5.6–11.1)
Sexual orientation		
Heterosexual	64.5 (59.3–69.5)	51.6 (46.8–56.3)
Homosexual	5.1 (3.6–7.0)	4.9 (3.7–6.5)
Bisexual	3.0 (2.2–4.1)	5.9 (4.6–7.4)
Other	1.3 (0.9–1.9)	1.8 (1.2–2.6)
No attraction	7.4 (5.9–9.3)	3.6 (2.5–5.1)
Uncertain	18.7 (15.9–21.8)	32.3 (28.5–36.4)
Mental health issues		
Depressive symptoms	6.3 (5.1–7.8)	4.9 (3.9–6.1)
Anxiety symptoms	27.5 (24.2–31.1)	19.7 (17.2–22.5)
Suicidal ideation	1.6 (1.2–2.2)	1.7 (1.2–2.4)

*Indonesia.* In Indonesia, 5.3% of adolescents identified as gender diverse, 5.9% as uncertain, and 88.8% as cisgender. In terms of sexual orientation, 9.4% of adolescents reported sexually diverse attractions, 7.4% reported no attraction to boys or girls, 18.7% were uncertain, and 64.5% reported heterosexual attractions. Overall, 6.3% of adolescents reported depressive symptoms, 27.5% reported anxiety symptoms, and 1.6% reported suicidal ideation.

*Vietnam.* In Vietnam, 5.9% of adolescents identified as gender diverse, 7.9% as uncertain, and 86.3% as cisgender. In terms of sexual orientation, 12.6% of adolescents reported sexually diverse attractions, 3.6% reported no attraction to boys or girls, 32.3% were uncertain, and 51.6% reported heterosexual attractions. Overall, 4.9% of adolescents reported depressive symptoms, 19.7% reported anxiety symptoms, and 1.7% reported suicidal ideation.

### Sociodemographic differences

Weighted sociodemographic characteristics and mental health issues by gender identity and sexual orientation across Indonesia and Vietnam are presented in Table 2 (gender identity) and 3 (sexual orientation).

**Table 2:** Sociodemographic characteristics and mental health issues by gender identity

	Indonesia				Vietnam			
	Cisgender	Gender diverse	Uncertain	*p* value	Cisgender	Gender diverse	Uncertain	*p* value
Primary caregiver-reported sex				0.037				0.012
Male	51.0 (48.7–53.3)	39.4 (31.8–47.5)	51.9 (43.2–60.5)		52.0 (49.7–54.4)	38.8 (31.0–47.3)	57.3 (48.3–65.9)	
Female	49.1 (46.7–51.3)	60.6 (52.5–68.2)	48.1 (39.5–56.8)		48.0 (45.6–50.3)	61.2 (52.7–69.0)	42.7 (34.1–51.7)	
Age group				0.112				0.750
12–14 years	52.9 (50.5–55.3)	57.6 (47.8–66.8)	62.0 (53.3–69.9)		51.8 (49.6–53.9)	50.4 (43.5–57.4)	54.3 (45.6–62.7)	
15–17 years	47.1 (44.7–49.5)	42.4 (33.2–52.3)	38.1 (30.1–46.7)		48.2 (46.1–50.4)	49.6 (42.6–56.5)	45.7 (37.3–54.4)	
Urbanicity				0.982				0.026
Rural	30.8 (22.6–40.4)	31.7 (19.2–47.5)	30.5 (18.9–45.3)		66.4 (61.3–71.2)	70.0 (59.0–79.0)	80.2 (68.4–88.4)	
Urban	69.2 (59.6–77.4)	68.3 (52.5–80.8)	69.5 (54.7–81.1)		33.6 (28.8–38.8)	30.0 (21.0–41.0)	19.8 (11.6–31.6)	
Wealth tertile				0.001				0.009
Low	33.2 (28.0–38.9)	38.4 (28.7–49.1)	48.0 (38.0–58.1)		31.4 (26.7–36.6)	39.7 (29.3–51.1)	49.9 (35.2–64.5)	
Medium	39.0 (35.4–42.7)	42.5 (33.3–52.2)	38.0 (29.2–47.6)		34.3 (30.4–38.4)	33.1 (25.2–42.2)	24.6 (16.5–35.0)	
High	27.8 (23.3–32.8)	19.1 (12.9–27.4)	14.0 (8.4–22.7)		34.3 (30.5–38.3)	27.2 (19.0–37.3)	25.6 (16.4–37.6)	
Sexual orientation				< 0.001				< 0.001
Heterosexual	72.1 (67.3–76.4)	7.6 (3.6–15.5)	0.0 (0.0–0.0)		58.3 (53.6–62.9)	16.6 (10.8–24.8)	0.0 (0.0–0.0)	
Sexually diverse	7.0 (5.1–9.4)	53.7 (43.4–63.8)	6.8 (3.4–13.1)		10.5 (8.6–12.8)	52.6 (43.2–61.7)	3.5 (1.8–6.7)	
No attraction	6.3 (4.9–8.0)	12.9 (6.8–23.3)	16.2 (10.7–23.7)		3.7 (2.6–5.3)	3.5 (1.5–7.9)	1.6 (0.6–3.9)	
Uncertain	14.7 (12.1–17.6)	25.7 (17.2–37.0)	77.1 (68.7–83.8)		27.4 (24.0–31.2)	27.3 (20.2–35.9)	94.9 (91.5–97.0)	
Mental health issues								
Depressive symptoms	5.7 (4.5–7.2)	15.3 (9.1–24.4)	8.8 (5.1–14.7)	< 0.001	4.5 (3.6–5.8)	8.3 (4.4–14.9)	6.0 (3.1–11.3)	0.146
Anxiety symptoms	25.7 (22.4–29.2)	40.1 (30.5–50.5)	44.6 (33.5–56.2)	< 0.001	19.1 (16.6–21.9)	28.0 (21.8–35.1)	21.7 (13.1–33.9)	0.149
Suicidal ideation	1.3 (0.9–1.9)	7.5 (3.8–14.4)	1.2 (0.3–4.8)	< 0.001	1.4 (1.0–2.1)	3.3 (1.4–7.5)	2.7 (1.1–6.7)	0.112

*Indonesia.* As shown in Table 2, a higher prevalence of gender diverse (38.4%) and uncertain (48.0%) Indonesian adolescents were from a lower wealth tertile than their cisgender peers (33.2%). There was also a noteworthy overlap between sexual and gender diversity: 53.7% of gender diverse adolescents reported sexually diverse attractions compared to 7.0% of cisgender adolescents and 6.8% of those who were uncertain. Gender diverse adolescents had a higher prevalence of depressive symptoms (15.3%) and suicidal ideation (7.5%) than cisgender (5.7% and 1.3%) or uncertain (8.8% and 1.2%) adolescents. In terms of anxiety symptoms, however, both gender diverse (40.1%) and uncertain (44.6%) adolescents had a comparably high prevalence as cisgender (25.7%) adolescents.

As shown in Table 3, a higher prevalence of Indonesian adolescents with sexually diverse attractions (63.2%), no attractions (68.8%), or uncertain attractions (66.9%) were in the younger age group than their heterosexual peers (47.1%). Sexually diverse adolescents had a higher prevalence of depressive symptoms (10.3%) than those with heterosexual attractions (4.8%), no attractions (6.9%), or uncertain attractions (8.9%). The prevalence of anxiety symptoms was highest among those with uncertain attractions (39.0%), followed by those with no attractions (36.1%), sexually diverse attractions (35.3%), and heterosexual attractions (20.7%). There were no significant differences in the prevalence of suicidal ideation between these four groups.

**Table 3:** Sociodemographic characteristics and mental health issues by sexual orientation

	Indonesia					Vietnam				
	Heterosexual	Sexually diverse	No attraction	Uncertain	*p* value	Heterosexual	Sexually diverse	No attraction	Uncertain	*p* value
Primary caregiver-reported sex					0.030					0.120
Male	52.5 (49.8–55.2)	46.8 (40.3–53.3)	42.0 (35.0–49.4)	50.2 (45.2–55.1)		52.9 (49.8–55.9)	46.7 (40.7–52.8)	41.8 (28.6–56.4)	53.9 (49.8–57.8)	
Female	47.5 (44.8–50.2)	53.2 (46.7–59.7)	58.0 (50.6–65.0)	49.8 (44.8–54.8)		47.1 (44.1–50.2)	53.3 (47.2–59.3)	58.2 (43.6–71.5)	46.1 (42.2–50.2)	
Age group					< 0.001					< 0.001
12–14 years	47.1 (44.0–50.3)	63.2 (56.8–69.2)	68.8 (61.4–75.4)	66.9 (62.4–71.2)		45.7 (42.3–49.1)	49.1 (43.7–54.5)	59.5 (49.6–68.6)	62.0 (57.5–66.3)	
15–17 years	52.9 (49.7–56.0)	36.8 (30.8–43.2)	31.2 (24.6–38.6)	33.1 (28.8–37.6)		54.3 (50.9–57.7)	50.9 (45.5–56.3)	40.5 (31.4–50.4)	38.0 (33.7–42.5)	
Urbanicity					0.401					0.016
Rural	31.6 (22.2–42.7)	39.7 (26.0–55.2)	27.6 (16.6–42.3)	27.9 (18.9–39.1)		62.4 (56.2–68.3)	76.8 (68.3–83.6)	68.2 (51.5–81.2)	71.7 (63.3–78.8)	
Urban	68.4 (57.3–77.8)	60.3 (44.8–74.0)	72.4 (57.7–83.4)	72.1 (60.9–81.2)		37.6 (31.7–43.9)	23.2 (16.4–31.7)	31.8 (18.8–48.5)	28.3 (21.2–36.7)	
Wealth tertile					0.018					< 0.001
Low	31.0 (25.2–37.6)	44.0 (34.3–54.2)	40.1 (30.1–51.0)	36.7 (30.1–43.8)		27.5 (22.2–33.6)	41.5 (33.3–50.3)	40.7 (28.6–54.1)	37.6 (30.2–45.5)	
Medium	39.1 (34.8–43.5)	37.9 (30.3–46.2)	38.2 (30.0–47.1)	39.8 (34.7–45.1)		33.5 (28.4–39.1)	32.3 (26.1–39.2)	36.8 (27.2–47.5)	33.5 (28.1–39.5)	
High	29.9 (24.4–36.0)	18.1 (11.9–26.6)	21.8 (15.4–29.8)	23.5 (18.3–29.6)		38.9 (33.8–44.3)	26.2 (19.9–33.6)	22.5 (13.3–35.4)	28.9 (23.9–34.5)	
Gender identity					< 0.001					< 0.001
Cisgender	99.4 (98.7–99.7)	66.2 (58.0–73.6)	78.8 (71.2–84.8)	71.0 (65.1–76.3)		98.1 (97.0–98.7)	72.9 (65.9–78.9)	91.0 (84.4–95.0)	74.1 (66.3–80.6)	
Gender diverse	0.6 (0.3–1.3)	30.0 (23.2–37.9)	9.5 (5.1–16.8)	7.3 (4.7–11.1)		1.9 (1.3–3.0)	25.2 (19.4–32.0)	5.9 (2.8–11.8)	5.1 (3.6–7.1)	
Uncertain	0.0 (0.0–0.0)	3.7 (1.9–7.4)	11.7 (7.7–17.5)	21.7 (17.1–27.0)		0.0 (0.0–0.0)	2.0 (1.0–4.0)	3.1 (1.3–7.4)	20.8 (14.8–28.4)	
Mental health issues										
Depressive symptoms	4.8 (3.6–6.4)	10.3 (6.8–15.4)	6.9 (4.0–11.8)	8.9 (6.2–12.6)	0.002	3.7 (2.6–5.1)	9.2 (5.6–14.7)	5.0 (2.1–11.2)	4.7 (3.2–6.8)	0.006
Anxiety symptoms	20.7 (17.2–24.7)	35.3 (27.4–44.1)	36.1 (28.6–44.3)	39.0 (33.2–45.1)	< 0.001	17.4 (14.5–20.7)	25.8 (20.8–31.6)	29.9 (17.6–46.2)	19.5 (15.5–24.2)	0.028
Suicidal ideation	1.2 (0.7–1.9)	3.4 (1.5–7.5)	2.3 (1.0–5.3)	2.2 (1.1–4.3)	0.064	1.0 (0.6–1.7)	3.0 (1.5–5.7)	2.8 (1.0–7.2)	2.2 (1.3–3.7)	0.027

*Vietnam.* A higher prevalence of gender diverse (39.7%) and uncertain (49.9%) Vietnamese adolescents came from a lower wealth tertile than their cisgender peers (31.4%; Table 2). As in Indonesia, there was a noteworthy overlap between sexual and gender diversity in Vietnam: 52.6% of gender diverse adolescents reported sexually diverse attractions compared to 10.5% of cisgender adolescents and 3.5% of those who were uncertain. There were no significant differences in the prevalence of depressive symptoms, anxiety symptoms, or suicidal ideation by gender identity.

A higher prevalence of Vietnamese adolescents with no attractions (59.5%) or uncertain attractions (62.0%) were in the younger age group than those with heterosexual attractions (45.7%) or sexually diverse attractions (49.1%; Table 3). There were also noteworthy differences in household wealth by sexual orientation, with the highest prevalence of sexually diverse adolescents coming from the lowest wealth tertile (41.5%). Sexually diverse adolescents had a higher prevalence of depressive symptoms (9.2%) than those with heterosexual attractions (3.7%), no attractions (5.0%), or uncertain attractions (4.7%). There were no significant differences in the prevalence of anxiety symptoms or suicidal ideation between these four groups.

### Regression models

Results from the unadjusted and adjusted weighted logistic regression models across Indonesia and Vietnam are presented in Table 4.

**Table 4:** Logistic regression models of the relationship between sexual and gender diversity and mental health issues

Indonesia						
	Depressive symptoms		Anxiety symptoms		Suicidal ideation	
	OR (95% CI)	aOR (95% CI)	OR (95% CI)	aOR (95% CI)	OR (95% CI)	aOR (95% CI)
Gender identity						
Cisgender	Ref.	Ref.	Ref.	Ref.	Ref.	Ref.
Gender diverse	2.98 (1.61–5.52)**	2.95 (1.56–5.59)**	1.94 (1.28–2.94)**	1.88 (1.24–2.85)**	6.34 (2.90–13.86)***	6.16 (2.73–13.89)***
Uncertain	1.59 (0.86–2.95)	1.66 (0.88–3.14)	2.33 (1.42–3.81)**	2.27 (1.37–3.75)**	0.93 (0.20–4.23)	0.85 (0.20–3.68)
Sexual orientation						
Heterosexual	Ref.	Ref.	Ref.	Ref.	Ref.	Ref.
Sexually diverse	2.29 (1.32–4.00)**	2.64 (1.51–4.62)**	2.09 (1.44–3.03)***	2.02 (1.41–2.89)***	3.03 (1.11–8.26)*	3.41 (1.25–9.29)*
No attraction	1.48 (0.80–2.73)	1.67 (0.91–3.07)	2.16 (1.45–3.23)***	2.20 (1.45–3.34)***	2.05 (0.80–5.23)	2.25 (0.87–5.78)
Uncertain	1.94 (1.25–3.03)**	2.22 (1.39–3.55)**	2.44 (1.74–3.43)***	2.52 (1.82–3.49)***	1.92 (0.79–4.66)	2.08 (0.86–5.03)

Vietnam						
Gender identity						
Cisgender	Ref.	Ref.	Ref.	Ref.	Ref.	Ref.
Gender diverse	1.89 (0.94–3.81)	1.78 (0.91–3.50)	1.65 (1.18–2.30)**	1.58 (1.13–2.22)**	2.31 (0.86–6.19)	2.14 (0.79–5.74)
Uncertain	1.35 (0.69–2.63)	1.35 (0.71–2.58)	1.17 (0.64–2.16)	1.15 (0.63–2.12)	1.92 (0.78–4.74)	2.00 (0.83–4.84)
Sexual orientation						
Heterosexual	Ref.	Ref.	Ref.	Ref.	Ref.	Ref.
Sexually diverse	2.68 (1.41–5.08)**	2.58 (1.36–4.92)**	1.65 (1.18–2.31)**	1.58 (1.14–2.19)**	3.03 (1.25–7.33)*	2.84 (1.13–7.14)*
No attraction	1.38 (0.55–3.50)	1.29 (0.50–3.34)	2.03 (0.97–4.23)	1.94 (0.93–4.03)	2.81 (0.92–8.58)	2.72 (0.87–8.47)
Uncertain	1.30 (0.83–2.04)	1.30 (0.82–2.06)	1.15 (0.83–1.59)	1.13 (0.81–1.57)	2.17 (1.15–4.11)*	2.25 (1.17–4.35)*

Note. * *p* < 0.05; ** *p* < 0.01; *** *p* < 0.001. Separate models considered gender identity and sexual orientation. Final models were adjusted for primary caregiver-reported sex, age, urbanicity, and wealth tertile

*Indonesia.* In Indonesia, gender diverse adolescents had nearly three times the odds of depressive symptoms in the past 12 months compared to their cisgender peers (aOR: 2.95; 95% CI: 1.56–5.59) after controlling for primary caregiver-reported sex, age, urbanicity, and wealth tertile. Gender diverse adolescents also had significantly higher odds of anxiety symptoms (aOR: 1.88; 95% CI: 1.24–2.85) and suicidal ideation (aOR: 6.16; 95% CI: 2.73–13.89). In addition, adolescents who were uncertain of their gender identity had over two times the odds of anxiety symptoms compared to their cisgender peers (aOR 2.27; 95% CI 1.37–3.75). In comparison to their heterosexual peers, sexually diverse adolescents had significantly higher odds of depressive symptoms (aOR: 2.64; 95% CI: 1.51–4.62), anxiety symptoms (aOR: 2.02; 95% CI: 1.41–2.89), and suicidal ideation (aOR: 3.41; 95% CI: 1.25–9.29). In the case of anxiety symptoms, the magnitude of this association was even higher among adolescents with uncertain attractions (aOR: 2.52; 95% CI: 1.82–3.49) or no attractions (aOR: 2.20; 95% CI: 1.45–3.34). Adolescents with uncertain attractions also had over two times the odds of depressive symptoms compared to their heterosexual peers (aOR: 2.22; 95% CI: 1.39–3.55).

*Vietnam.* In Vietnam, gender diverse adolescents only had higher odds of anxiety symptoms in the past 12 months compared to their cisgender peers (aOR: 1.58; 95% CI: 1.13–2.22) after controlling for covariates. By contrast, in comparison to their heterosexual peers, sexually diverse adolescents had significantly increased odds of depressive symptoms (aOR: 2.58; 95% CI: 1.36–4.92), anxiety symptoms (aOR: 1.58; 95% CI: 1.14–2.19), and suicidal ideation (aOR: 2.84; 95% CI 1.13–7.14). In addition, adolescents with uncertain attractions had over two times the odds of suicidal ideation (aOR: 2.25; 95% CI: 1.17–4.35) when compared to their heterosexual peers.

*Supplemental analyses.* Results from the supplemental analyses, which considered full diagnosis of major depressive disorder and anxiety disorders (generalized anxiety disorder, social phobia, and any anxiety disorder) according to DSM-5 criteria, are presented in Table 5. In Indonesia, there was evidence for statistically significant associations between gender diversity and major depressive disorder (aOR: 4.71; 95% CI: 1.73–12.86), any anxiety disorder (aOR: 3.50; 95% CI: 1.64–7.45), generalized anxiety disorder (aOR: 4.06; 95% CI: 1.65–9.98), and social phobia (aOR: 3.29; 95% CI: 1.32–8.21). Similar patterns were found for the relationships between sexual diversity and both major depressive disorder (aOR: 3.25; 95% CI: 1.27–8.31) and any anxiety disorder (aOR: 2.63; 95% CI: 1.22–5.60). In Vietnam, there was evidence for statistically significant associations between gender diversity and both any anxiety disorder (aOR: 2.55; 95% CI: 1.21–5.40) and generalized anxiety disorder (aOR: 3.51; 95% CI: 1.61–7.63). However, while there was evidence for a relationship between sexual diversity and major depressive disorder (aOR: 4.12; 95% CI: 1.34–12.68), the association with anxiety disorders lacked statistical significance.

**Table 5:** Logistic regression models of the relationship between sexual and gender diversity and mental disorders

Indonesia				
	Major depressive disorder	Any anxiety disorder	Generalized anxiety disorder	Social phobia
	aOR (95% CI)	aOR (95% CI)	aOR (95% CI)	aOR (95% CI)
Gender identity				
Cisgender	Ref.	Ref.	Ref.	Ref.
Gender diverse	4.71 (1.73–12.86)**	3.50 (1.64–7.45)**	4.06 (1.65–9.98)**	3.29 (1.32–8.21)*
Uncertain	2.96 (1.00–8.74)	2.78 (1.33–5.78)**	5.20 (2.30–11.77)**	2.13 (0.98–4.66)
Sexual orientation				
Heterosexual	Ref.	Ref.	Ref.	Ref.
Sexually diverse	3.25 (1.27–8.31)*	2.63 (1.22–5.60)*	2.05 (0.48–8.79)	2.26 (0.94–5.43)
No attraction	1.33 (0.39–4.55)	2.53 (1.21–5.29)*	3.97 (1.63–9.65)**	1.78 (0.74–4.32)
Uncertain	1.76 (0.70–4.40)	2.00 (1.11–3.62)*	3.62 (1.86–7.07)***	1.76 (0.85–3.64)

Vietnam				
Gender identity				
Cisgender	Ref.	Ref.	Ref.	Ref.
Gender diverse	2.62 (0.89–7.67)	2.55 (1.21–5.40)*	3.51 (1.61–7.63)**	1.54 (0.31–7.57)
Uncertain	2.65 (0.94–7.45)	1.40 (0.54–3.68)	1.62 (0.55–4.76)	1.25 (0.31–5.08)
Sexual orientation				
Heterosexual	Ref.	Ref.	Ref.	Ref.
Sexually diverse	4.12 (1.34–12.68)*	1.32 (0.52–3.36)	1.42 (0.46–4.43)	1.29 (0.46–3.62)
No attraction	0.27 (0.03–2.22)	0.86 (0.20–3.71)	1.20 (0.26–5.47)	-
Uncertain	1.13 (0.43–2.98)	1.21 (0.61–2.41)	1.24 (0.53–2.92)	1.59 (0.64–3.97)

Note. * *p* < 0.05; ** *p* < 0.01; *** *p* < 0.001. Separate models considered gender identity and sexual orientation. Models were adjusted for primary caregiver-reported sex, age, urbanicity, and wealth tertile

## Discussion

To our knowledge, this is the first study to use nationally representative data to explore sexual and gender diversity and mental health among adolescents in Indonesia and Vietnam. We found that in both countries, a strikingly high percentage of adolescents reported gender diverse identities (5.3% Indonesia, 5.9% Vietnam) and sexually diverse orientations (9.4% Indonesia, 12.6% Vietnam). Despite the widespread marginalization of SGD populations in these settings [43, 52], this prevalence is generally higher than what has previously been documented across other Asian contexts, particularly in the case of gender diversity. For example, according to recent nationally representative surveys that used similar methodologies for determining gender identity and sexual orientation, 2.5% of adolescents in Thailand identified as gender diverse [13] while 4.1% of adolescents in China indicated sexually diverse attractions [63]. Similarly, while it was not nationally representative, a large-scale study of young people living in three Asian cities found that 3.9% of young people in Hanoi identified as sexually diverse, although prevalence was significantly higher in Shanghai (10.4%) and Taipei (8.6%) [33]. Notably, the prevalence of sexually diverse adolescents in NAMHS is comparable to those found in population-based samples from North America and Europe [8, 9, 14, 64].

In both Indonesia and Vietnam, there were also substantial numbers of adolescents who reported being uncertain of their gender identity (5.9% Indonesia, 7.9% Vietnam) or sexual orientation (18.7% Indonesia, 32.3% Vietnam). It is important to note that these numbers may not accurately reflect the underlying prevalence of adolescents questioning their gender identity or sexual orientation. Adolescents were categorized as “uncertain” if they indicated “don’t know” in answer to the relevant survey questions, but it is likely that this response also included those who did not fully understand the question or who were reluctant to answer due to social stigma [65]. Despite this possibility, emerging research points to the importance of studying adolescents who are questioning, unsure of, or exploring their gender or sexual identity due to the centrality of identity formation during this developmental period [66]. In the current study, a higher prevalence of those who reported being uncertain about their attractions were in the younger age group (12–14 years old) across both countries. This is consistent with this developmental stage, and matches patterns that have been documented elsewhere [9, 63]. These age-related patterns also extended to adolescents who indicated that they were not attracted to either boys or girls (7.4% Indonesia, 3.6% Vietnam). Given this finding, we suspect that this group reflects maturational differences within the sample, and largely includes adolescents who are not yet interested in romantic relationships [67]. It is also possible that this group includes those who might later identify as asexual and/or aromantic [68]; this is noteworthy given the lack of existing visibility around these identity labels in the study countries.

Notable sociodemographic differences also arose in terms of household wealth. In both Indonesia and Vietnam, a higher prevalence of gender diverse adolescents came from a lower wealth tertile than their cisgender peers, and there were even greater disparities in household wealth between those who were uncertain about their gender identity compared to those who were cisgender. In addition, in Vietnam, a higher prevalence of sexually diverse adolescents came from a lower wealth tertile than their heterosexual peers. It is challenging to explain these patterns based on available data. In terms of those who reported uncertain gender identities, it is possible that greater household wealth corresponds with greater exposure to information regarding gender diversity (e.g., through internet access), thereby decreasing levels of uncertainty. However, this argument cannot be used to explain the relationship between greater sexual diversity and lower household wealth in Vietnam. It is clear that further research is required in both countries in order to tease apart these patterns and better understand the implications for reaching SGD adolescents.

It is also important to discuss the substantial overlap between sexual and gender diversity in these data. In both Indonesia and Vietnam, over half of gender diverse adolescents reported sexually diverse attractions. This is not a novel finding as similarly high percentages have been documented among gender diverse adolescents across other settings [18, 69, 70]. It is noteworthy, however, given prior evidence which suggests that adolescents at the intersection of sexual and gender diversity may have a particularly heightened risk of adverse mental health outcomes compared to those who identify as only sexually diverse or gender diverse [18, 71]. Due to the almost complete lack of available data around both gender identity and sexual orientation among adolescents in Indonesia and Vietnam, we chose to analyse these populations separately to establish a baseline for future comparisons. In addition, sexual orientation was somewhat collinear with gender identity in our study due to the operationalization of these variables, introducing the potential for false inferences. That said, our findings suggest that future studies in both countries should explore the unique challenges faced by adolescents with intersecting SGD identities.

Overall, we found robust associations between sexual diversity and mental health issues in Indonesia and Vietnam. In both countries, the magnitude of this association was greatest for suicidal ideation (Indonesia aOR: 3.41; Vietnam aOR: 2.84), although these estimates had wide confidence intervals given that they were based on small numbers of adolescents. This aligns with a substantial body of literature on suicidal behaviours among sexually diverse adolescents worldwide [4–6], and underscores the necessity of targeting suicide prevention efforts in Indonesia and Vietnam towards these groups. In addition to suicidal ideation, sexually diverse adolescents in both countries also had an increased risk of both depressive symptoms and anxiety symptoms, which again matches findings from other settings [2, 3]. Notably, in Indonesia, we found that the relationship with anxiety symptoms was even stronger for those who had no attractions as well as those who were uncertain about their attractions. Those with uncertain identities also exhibited increased odds of depressive symptoms in Indonesia and suicidal ideation in Vietnam. Similar patterns have emerged in other environments [9, 66, 72], suggesting that those who are struggling with understanding their sexual orientation at this age may need particular support.

There was more cross-country variation in the relationship between gender diversity and mental health issues. In Indonesia, there was evidence for associations between gender diversity and depressive symptoms (aOR: 2.95), anxiety symptoms (aOR: 1.88) and suicidal ideation (aOR: 6.16), and these patterns matched what was observed for sexual diversity. In Vietnam, however, there was only evidence for a relationship between gender diversity and anxiety symptoms (aOR: 1.58). While the findings from Indonesia are consistent with emerging evidence on mental health among gender diverse adolescents worldwide [12–18], the absence of associations beyond anxiety in Vietnam is worth further consideration. One possible explanation is that we lacked the power to detect a significant relationship given the relatively small percentage of gender diverse adolescents in Vietnam (5.9%) alongside the low prevalence of both depressive symptoms (4.9%) and suicidal ideation (1.7%). An alternative hypothesis relates to a general lack of awareness around gender diversity in Vietnam, with non-normative gender identities traditionally conceptualized as homosexuality, thereby rendering gender diverse populations invisible [49, 73]. It is conceivable that this would lead to reduced minority stress among adolescents who self-identity as gender diverse, but who do not outwardly present as gender non-conforming.

Findings from the current study must be interpreted in light of several limitations. Most importantly, there is the potential for underreporting in both the independent and dependent variables due to the stigma associated with SGD identities [43, 51, 52] and mental health issues [74, 75] in Indonesia and Vietnam. For example, one qualitative study in Indonesia documented concerns around growing hostility towards SGD individuals countrywide [43], which might deter adolescents from disclosing diverse identities. Likewise, large-scale survey research in Vietnam has found evidence of very low tolerance for homosexuality among young people [52]. It is important to note, however, that all SGD-related questions were self-administered in order to increase the accuracy of responses related to topics that may be considered particularly sensitive. It is also possible that the DSM-5-based mental health instrument did not capture important culturally specific dimensions of psychological distress in Indonesia and Vietnam. For instance, somatic symptom presentations have been identified as central to anxiety and depression in many Asian contexts, including among Indonesian and Vietnamese populations [76–79]. In addition, the cross-sectional nature of NAMHS precludes any type of causal inference. There may also have been sampling bias due to the exclusion of adolescents who lived in the household less than 50% of the time: studies in other settings have reported disproportionately higher rates of homelessness among SGD adolescents [80]. Further, while the current study suggests an association between sexual and gender diversity and mental health issues among adolescents, we did not assess specific minority stressors (e.g., family rejection, identity-based victimization) which may compromise mental health among SGD adolescents. To better inform mental health prevention and intervention efforts in Indonesia and Vietnam, future studies should explore the mental health risks associated with experiences of stigma, discrimination, and victimization among SGD adolescents in both countries.

An additional limitation relates to the measurement of both gender identity and sexual orientation. In the case of gender identity, the recommended best practice is to follow a two-step method in which sex assigned at birth is compared with current gender identity [81]. While we followed a similar approach in the current study, we lacked data on sex assigned at birth and thus had to rely on primary caregiver-reported sex instead. Although it is likely that most primary caregivers did report on their adolescent’s sex assigned at birth, it is theoretically possible that some primary caregivers instead reported on their adolescent’s current gender identity. In these cases, adolescents would not have been captured as gender diverse because their caregiver’s report would have matched their self-report. It is important to emphasize, however, that such misreporting would theoretically bias any associations with mental health issues towards the null. In the case of sexual orientation, the current study only included a single question assessing romantic/sexual attraction. Sexual orientation, however, is a multidimensional construct which encompasses identity, attraction, and behaviour [82]. A number of studies among adolescents have found that the prevalence of sexual diversity, as well as associated health issues, can vary greatly based on whether identity, attraction, or behaviour is assessed [83–86]. As such, it is suggested that surveys measure multiple dimensions of sexual orientation in order to accurately capture complexity within sexually diverse populations [65]. In addition, given the high number of adolescents who endorsed “don’t know” or “prefer not to say” in relation to their gender identity or romantic/sexual attraction, measure development should focus on ensuring that questions are culturally appropriate and comprehensible to younger age groups.

## Conclusions

This study found that a considerable proportion of adolescents in both Indonesia and Vietnam report gender diverse identities or sexually diverse orientations. In addition, SGD adolescents across both countries are at increased risk of experiencing a range of mental health issues, including depression, anxiety, and suicidal ideation. Future research should build on these findings by developing more comprehensive measurement approaches, exploring key sociodemographic variations, and investigating the role of minority stress in driving mental health challenges among SGD adolescents. Overall, findings from this study speak to the importance of measuring sexual and gender diversity in national mental health surveys to better inform targeted policies and programs. In Indonesia and Vietnam, the needs of SGD adolescents have historically been excluded from both general public health strategies as well as specific mental health efforts. Results from the current study highlight the necessity of appropriate, culturally safe mental health and psychosocial support services that can help SGD adolescents as they navigate this critical period of identity development. Further, it is clear that there is a need for targeted support for adolescents who are uncertain about their gender identity or sexual orientation. Finally, upstream efforts in both countries should focus on reducing stigma and discrimination, thus contributing to greater safety and well-being among these young people.

## Electronic supplementary material

Supplementary Material 1.

## Data Availability

The NAMHS datasets are co-owned by the University of Queensland and each respective in-country lead organisation (K-NAMHS: APHRC and UQ; I-NAMHS: UGM and UQ; V-NAMHS: IOS and UQ). Currently, these datasets or analysis of these datasets are available for collaborative work on request to the relevant data owners following an established protocol. Work is currently underway to convert the NAMHS datasets into public use datasets, allowing for wide use of these datasets while ensuring protection of participant privacy, adherence to country-specific legislation, and appropriate use of data. This includes development of accompanying meta-data, inclusive of a codebook, technical manual, and analysis files. The expected launch date for these public use datasets and accompanying meta-data is 2026, with hosting mechanisms currently under development in line with country legislation and ethical requirements.

## Abbreviations

SGD: Sexually and gender diverse
LMICs: Low- and middle-income countries
MSM: Men who have sex with men
NAMHS: National Adolescent Mental Health Surveys
I-NAMHS: Indonesia - National Adolescent Mental Health Survey
V-NAMHS: Viet Nam Adolescent Mental Health Survey
DISC-5: Diagnostic Interview Schedule for Children, Version 5
DSM-5: Diagnostic and Statistical Manual of Mental Disorders, Fifth Edition

## Declarations

### About this supplement

This article has been published as part of Child and Adolescent Psychiatry and Mental Health, Volume 19 Supplement S1, 2025: Findings from the National Adolescent Mental Health Surveys: Factors influencing adolescent mental health in Kenya, Indonesia, and Vietnam. The full contents of the supplement are available at https://capmh.biomedcentral.com/articles/supplements/volume-19-supplement-1.
