# Supplementary material for: Mental health among sexually and gender diverse adolescents in Indonesia and Vietnam: Results from the National Adolescent Mental Health Surveys
Source: Child Adolesc Psychiatry Ment Health. 2025 Jul 31;19(Suppl 1):82. doi: 10.1186/s13034-025-00921-5 (PMC12312252; doi:10.1186/s13034-025-00921-5)
Supplement: Supplementary file 1 — Supplementary Material 1. [file 13034_2025_921_MOESM1_ESM.docx]

**Supplemental Tables**

**Table S1.** Sociodemographic characteristics and mental health issues by country (unweighted numbers and weighted percentages)

|  | **Indonesia (N = 4,300)** | **Vietnam (N = 4,376)** |
| --- | --- | --- |
|  | N (%) | N (%) |
| **Primary caregiver-reported sex** |  |  |
| Male | 2,106 (50.5) | 2,292 (51.9) |
| Female | 2,194 (49.5) | 2,084 (48.1) |
| **Age group** |  |  |
| 12-14 | 2,251 (53.9) | 2,382 (51.9) |
| 15-17 | 2,049 (46.1) | 1,994 (48.1) |
| **Urbanicity** |  |  |
| Rural | 1,392 (30.8) | 2,199 (67.8) |
| Urban | 2,908 (69.2) | 2,177 (32.2) |
| **Gender identity** |  |  |
| Cisgender | 3,774 (88.8) | 3,792 (86.3) |
| Transgender | 107 (2.6) | 127 (2.6) |
| Non-binary | 79 (2.3) | 102 (2.5) |
| Other | 14 (0.4) | 24 (0.8) |
| Uncertain | 247 (5.9) | 257 (7.9) |
| **Sexual orientation** |  |  |
| Heterosexual | 2,584 (64.5) | 2,279 (51.6) |
| Homosexual | 189 (5.1) | 193 (4.9) |
| Bisexual | 112 (3.0) | 257 (5.9) |
| Other | 43 (1.3) | 62 (1.8) |
| No attraction | 288 (7.4) | 117 (3.6) |
| Uncertain | 722 (18.7) | 1,137 (32.3) |
| **Mental health issues** |  |  |
| Depressive symptoms | 256 (6.3) | 202 (4.9) |
| Anxiety symptoms | 1,268 (27.5) | 781 (19.7) |
| Suicidal ideation | 85 (1.6) | 74 (1.7) |

*Note.* All numbers are unweighted whereas percentages have been adjusted for population weights.

**Table S2.** Sociodemographic characteristics and mental health issues by gender identity (unweighted numbers and weighted percentages)

|  | **Indonesia** | | | | **Vietnam** | | | |
| --- | --- | --- | --- | --- | --- | --- | --- | --- |
|  | Cisgender  (n=3,774) | Gender diverse  (n=200) | Uncertain  (n=247) | *p* value | Cisgender  (n=3,792) | Gender diverse  (n=253) | Uncertain  (n=257) | *p* value |
| **Primary caregiver-reported sex** |  |  |  | 0.037 |  |  |  | 0.012 |
| Male | 1,856 (51.0) | 87 (39.4) | 124 (51.9) |  | 2,001 (52.0) | 107 (38.8) | 142 (57.3) |  |
| Female | 1,918 (49.1) | 113 (60.6) | 123 (48.1) |  | 1,791 (48.0) | 146 (61.2) | 115 (42.7) |  |
| **Age group** |  |  |  | 0.112 |  |  |  | 0.750 |
| 12-14 | 1,951 (52.9) | 109 (57.6) | 142 (62.0) |  | 2,041 (51.8) | 146 (50.4) | 152 (54.3) |  |
| 15-17 | 1,823 (47.1) | 91 (42.4) | 105 (38.1) |  | 1,751 (48.2) | 107 (49.6) | 105 (45.7) |  |
| **Urbanicity** |  |  |  | 0.982 |  |  |  | 0.026 |
| Rural | 1,206 (30.8) | 73 (31.7) | 85 (30.5) |  | 1,865 (66.4) | 131 (70.0) | 163 (80.2) |  |
| Urban | 2,568 (69.2) | 127 (68.3) | 162 (69.5) |  | 1,927 (33.6) | 122 (30.0) | 94 (19.8) |  |
| **Wealth tertile** |  |  |  | 0.001 |  |  |  | 0.009 |
| Low | 1,226 (33.2) | 75 (38.4) | 120 (48.0) |  | 886 (31.4) | 70 (39.7) | 96 (49.9) |  |
| Medium | 1,535 (39.0) | 88 (42.5) | 95 (38.0) |  | 1,137 (34.3) | 83 (33.1) | 76 (24.6) |  |
| High | 1,013 (27.8) | 37 (19.1) | 32 (14.0) |  | 1,769 (34.3) | 100 (27.2) | 85 (25.6) |  |
| **Sexual orientation** |  |  |  | <0.001 |  |  |  | <0.001 |
| Heterosexual | 2,564 (72.1) | 20 (7.6) | 0 (0.0) |  | 2,230 (58.3) | 49 (16.6) | 0 (0.0) |  |
| Sexually diverse | 238 (7.0) | 89 (53.7) | 15 (6.8) |  | 376 (10.5) | 127 (52.6) | 8 (3.5) |  |
| No attraction | 222 (6.3) | 27 (12.9) | 29 (16.2) |  | 104 (3.7) | 7 (3.5) | 6 (1.6) |  |
| Uncertain | 514 (14.7) | 50 (25.7) | 145 (77.1) |  | 870 (27.4) | 57 (27.3) | 198 (94.9) |  |
| **Mental health issues** |  |  |  |  |  |  |  |  |
| Depressive symptoms | 203 (5.7) | 25 (15.3) | 24 (8.8) | <0.001 | 158 (4.5) | 19 (8.3) | 20 (6.0) | 0.146 |
| Anxiety symptoms | 1,056 (25.7) | 79 (40.1) | 106 (44.6) | <0.001 | 637 (19.1) | 73 (28.0) | 63 (21.7) | 0.149 |
| Suicidal ideation | 67 (1.3) | 13 (7.5) | 3 (1.2) | <0.001 | 54 (1.4) | 10 (3.3) | 7 (2.7) | 0.112 |

*Note.* All numbers are unweighted whereas percentages have been adjusted for population weights.

**Table S3.** Sociodemographic characteristics and mental health issues by sexual orientation (unweighted numbers and weighted percentages)

|  | **Indonesia** | | | | | **Vietnam** | | | | |
| --- | --- | --- | --- | --- | --- | --- | --- | --- | --- | --- |
|  | Heterosexual  (n=2,584) | Sexually diverse  (n=344) | No attraction  (n=288) | Uncertain  (n=722) | *p* value | Heterosexual (n=2,279) | Sexually diverse (n=512) | No attraction (n=117) | Uncertain (n=1,137) | *p* value |
| **Primary caregiver-reported sex** |  |  |  |  | 0.030 |  |  |  |  | 0.120 |
| Male | 1,302 (52.5) | 158 (46.8) | 121 (42.0) | 367 (50.2) |  | 1,228 (52.9) | 254 (46.7) | 48 (41.8) | 594 (53.9) |  |
| Female | 1,282 (47.5) | 186 (53.2) | 167 (58.0) | 355 (49.8) |  | 1,051 (47.1) | 258 (53.3) | 69 (58.2) | 543 (46.1) |  |
| **Age group** |  |  |  |  | <0.001 |  |  |  |  | <0.001 |
| 12-14 | 1,199 (47.1) | 216 (63.2) | 194 (68.8) | 455 (66.9) |  | 1,089 (45.7) | 273 (49.1) | 71 (59.5) | 761 (62.0) |  |
| 15-17 | 1,385 (52.9) | 128 (36.8) | 94 (31.2) | 267 (33.1) |  | 1,190 (54.3) | 239 (50.9) | 46 (40.5) | 376 (38.0) |  |
| **Urbanicity** |  |  |  |  | 0.401 |  |  |  |  | 0.016 |
| Rural | 822 (31.6) | 127 (39.7) | 90 (27.6) | 246 (27.9) |  | 1,010 (62.4) | 313 (76.8) | 59 (68.2) | 633 (71.7) |  |
| Urban | 1762 (68.4) | 217 (60.3) | 198 (72.4) | 476 (72.1) |  | 1,269 (37.6) | 199 (23.2) | 58 (31.8) | 504 (28.3) |  |
| **Wealth tertile** |  |  |  |  | 0.018 |  |  |  |  | <0.001 |
| Low | 775 (31.0) | 146 (44.0) | 110 (40.1) | 284 (36.7) |  | 460 (27.5) | 160 (41.5) | 37 (40.7) | 325 (37.6) |  |
| Medium | 1,049 (39.1) | 136 (37.9) | 118 (38.2) | 289 (39.8) |  | 640 (33.5) | 163 (32.3) | 41 (36.8) | 366 (33.5) |  |
| High | 760 (29.9) | 62 (18.1) | 60 (21.8) | 149 (23.5) |  | 1,179 (38.9) | 189 (26.2) | 39 (22.5) | 44 (28.9) |  |
| **Gender identity** |  |  |  |  | <0.001 |  |  |  |  | <0.001 |
| Cisgender | 2,564 (99.4) | 238 (66.2) | 222 (78.8) | 514 (71.0) |  | 2,230 (98.1) | 376 (72.9) | 104 (91.0) | 870 (74.1) |  |
| Gender diverse | 20 (0.6) | 89 (30.0) | 27 (9.5) | 50 (7.3) |  | 49 (1.9) | 127 (25.2) | 7 (5.9) | 57 (5.1) |  |
| Uncertain | 0 (0.0) | 15 (3.7) | 29 (11.7) | 145 (21.7) |  | 0 (0.0) | 8 (2.0) | 6 (3.1) | 198 (20.8) |  |
| **Mental health issues** |  |  |  |  |  |  |  |  |  |  |
| Depressive symptoms | 117 (4.8) | 30 (10.3) | 24 (6.9) | 51 (8.9) | 0.002 | 76 (3.7) | 39 (9.2) | 7 (5.0) | 58 (4.7) | 0.006 |
| Anxiety symptoms | 610 (20.7) | 124 (35.3) | 104 (36.1) | 277 (39.0) | <0.001 | 326 (17.4) | 130 (25.8) | 32 (29.9) | 228 (19.5) | 0.028 |
| Suicidal ideation | 39 (1.2) | 11 (3.4) | 9 (2.3) | 21 (2.2) | 0.064 | 25 (1.0) | 15 (3.0) | 5 (2.8) | 25 (2.2) | 0.027 |

*Note.* All numbers are unweighted whereas percentages have been adjusted for population weights.
